# Treatment of patients with multiple myeloma progressing on frontline-therapy with lenalidomide

**DOI:** 10.1038/s41408-019-0200-1

**Published:** 2019-03-20

**Authors:** Philippe Moreau, Elena Zamagni, Maria-Victoria Mateos

**Affiliations:** 1 University Hospital Hotel-Dieu, Nantes, France; 2 Istituto Seragnoli, Bologna, Italy; 3 University Hospital, Salamanca, Spain

## Abstract

Over the last years, there has been great progress in the treatment of multiple myeloma with many new agents and combinations having been approved and being now routinely incorporated into treatment strategies. As a result, patients are experiencing benefits in terms of survival and better tolerance. However, the multitude of treatment options also presents a challenge to select the best options tailored to the specific patient situation. Lenalidomide is increasingly being used as part of frontline therapy in newly diagnosed multiple myeloma. This agent is typically administered until disease progression. It is currently unclear, how to best manage patients, who relapse while receiving lenalidomide as part of their frontline treatment. We conducted a review to summarize the available evidence in this setting. Our summary shows that there are very few data from current trials testing new combinations based on carfilzomib, pomalidomide, or daratumumab that address this specific patient population. Our review is aimed to summarize the available evidence to assist treatment decision making and to raise awareness of this lack of data to encourage further analyses and the incorporation of sequencing questions in future trial designs.

## Introduction

The treatment of multiple myeloma (MM) has changed dramatically in the past decade with the introduction of new drugs into therapeutic strategies, both in the frontline and in the relapse settings. These drugs have been incorporated into national and international clinical guidelines, and have transformed our approach to the treatment of patients with MM^1,2^.

With the availability of at least 6 different classes of approved agents, i.e., alkylators, steroids, proteasome inhibitors (PIs), immunomodulatory agents (IMiDs), histone deacetylase inhibitors (DACIs) and monoclonal antibodies (MoAbs) that can be combined in doublet, triplet or even quadruplet regimens, and used together with or without high-dose therapy and autologous stem cell transplantation (ASCT), or in some cases as continuous treatment, the choice of the optimal strategy at diagnosis and at relapse represents a challenge for physicians. Also, problematic is the lack of trials addressing important questions, such as the integration of the first salvage regimen into the assessment of front-line therapies in order to define optimal sequencing strategies and to evaluate progression-free survival 2 (PFS2) as an important end point in homogeneous patient populations. Furthermore, there is a substantial lack of pre-planned large subgroup analyses in most of the recently conducted trials, which led to the approval of various triplet combinations at relapse, concerning issues, such as treatment of refractory disease versus treatment of relapse occurring after a treatment-free interval, biochemical versus symptomatic relapse, relapse after 1 prior line versus more advanced disease, high-risk versus standard risk cytogenetics, etc^3^.

At the time of the first relapse, the treatment choice is influenced by many patient- and disease-related factors, such as age, cytogenetics, pre-existing toxicities, aggressiveness of the relapse, but mostly by the type of frontline treatment and the response to the previous therapy^4^. Since frontline lenalidomide given until progression is becoming one of the preferred options to treat patients with MM^1,2^, it is important to carefully evaluate salvage regimens that can be proposed at the time of first progression on lenalidomide. The aim of this manuscript is to review current available data for the treatment of patients progressing on lenalidomide provided as first-line therapy.

### Lenalidomide use as frontline therapy and lenalidomide-refractoriness

Lenalidomide is increasingly being used as part of frontline therapy in newly diagnosed MM (NDMM). In patients treated with upfront ASCT, lenalidomide single agent at low dose is approved as maintenance therapy until progression^5^. In a meta-analysis of 3 randomized clinical trials using primary-source patient-level data comparing lenalidomide maintenance versus placebo or observation, a significant benefit in PFS and overall survival (OS) were demonstrated with lenalidomide maintenance after ASCT^5^. In patients with previously untreated NDMM, who are not eligible for ASCT, lenalidomide is also approved in combination with low-dose dexamethasone (Rd) until disease progression, based on the results of the randomized FIRST study^6^. In the final analysis of this trial, treatment with continuous Rd significantly improved survival outcomes versus melphalan-prednisone-thalidomide (MPT), supporting continuous Rd as a standard of care for patients with transplant-ineligible NDMM^7^. In addition, in the prospective SWOG0777 trial, which enrolled patients with NDMM, who were not intended to undergo immediate ASCT, Rd was compared to Rd plus bortezomib (VRd)^8^. The addition of bortezomib resulted in significantly improved PFS and OS. Upon completion of induction, all patients received ongoing maintenance with Rd until progression. The regimen of VRd followed by Rd until progression is now also recommended by several guidelines as upfront therapy for transplant ineligible patients^1,2^.

Overall, at the present time, a high proportion of patients with NDMM receive frontline lenalidomide until progression, either as single-agent at low dose (10 or 15 mg/day) or at the full dose in combination with low-dose weekly dexamethasone, meaning that their disease will progress while on therapy. However, in this setting, there is no consensus regarding the definition of lenalidomide-refractoriness. While in some cases, disease progression will be characterized by a symptomatic relapse with lytic lesions or other symptoms of end-organ damage, in other cases, patients will experience a biochemical relapse only as defined by the IMWG criteria, with an increase of 25% or more in monoclonal protein, with an absolute increase of at least 0.5 g/dL, without symptoms of end-organ damage. The first group of patients will require the immediate initiation of an alternative therapy. For the second group of patients, with a slow rise in the paraprotein level, some investigators consider that increasing the dose of lenalidomide from 10–15 mg/day to 25 mg/day and adding dexamethasone before the occurrence of symptoms may be effective, at least in standard-risk patients when relapse occurs during low-dose lenalidomide maintenance. In fact, no clear data are available supporting this approach. Among the three trials included in the meta-analysis that supports the use of lenalidomide maintenance^9–11^, salvage therapies following progression on lenalidomide are listed in two of them^10,11^, but the outcome of patients treated with an increased dose of lenalidomide and the addition of dexamethasone is unknown. These data are also lacking in the recent Myeloma XI trial, which showed PFS and OS benefits with lenalidomide maintenance in transplant eligible patients^12^. Consequently, the majority of experts consider that salvage therapy consisting of full-dose lenalidomide plus dexamethasone for patients progressing on low-dose lenalidomide maintenance is insufficient, and these patients are often considered lenalidomide-refractory. These patients were also rightly excluded from recent randomized phase 3 trials testing Rd vs Rd plus a third agent (either a PI, [carfilzomib^13^, KRd, or ixazomib^14^, IRd] or a mAb, [elotuzumab^15^, Elo-Rd, or daratumumab^16^, DRd]). The exact role of lenalidomide-based triplet combinations in patients refractory to lenalidomide is unknown, however, most likely they are suboptimal, and these regimens are therefore rarely used in this setting. The only study available showing that the addition of a third agent to lenalidomide and steroids may rescue lenalidomide-refractory disease is a phase 1/2 trial reported by the HOVON group^17^. In 67 patients (median three prior lines of therapy), Nijhof et al. showed that the addition of continuous low-dose oral cyclophosphamide (50 mg/d) to 25 mg lenalidomide and prednisone (REP regimen) induced a 67% response rate, with a median PFS and OS of 12.1 and 29 months, respectively, in lenalidomide-refractory patients (Table 1)^17^. Interestingly, the majority of lenalidomide-refractory cases enrolled in the study had progressed while receiving lenalidomide at 25 mg plus dexamethasone. Unfortunately, data for patients progressing on frontline lenalidomide are not reported. Nevertheless, these remarkable results, which show that low-dose metronomic oral cyclophosphamide may revert lenalidomide resistance, needs further evaluation.

**Table 1:** Sub-analysis of patients with lenalidomide-refractory disease in phase 3 and 1b/2 trials: number of enrolled patients and PFS outcomes

Phase 3 trials						
	Endeavor^21^		Castor^23^		Optimissm^28^	
	Kd	Vd	DVd	Vd	PVd	Vd
Len-refractory to any prior line, n =	51	45	60	81	120	118
Median PFS months	8.6	6.6	7.8	4.9	9.5	5.6
Len-refractory to 1 prior line, n =	UK	UK	UK	UK	64	65
Median PFS months	UK	UK	UK	UK	17.8	9.5
*Phase 1b/2 trials*						
Trial	REP^17^	MMY1001^24^	MMY1001^24^	MM014^32^	EMN^36^	
Regimen	Len-Cy-Pred	DKd	DPd	DPd	KPd	
Len-refractory to any prior line, n =	67	51	92	84	60	
Median PFS months	12.1	12-month: 65%	10.1	9-month: 86%	18	
Len-refractory to 1 prior line, n =	UK	6	UK	UK	60	
Median PFS months	UK	UK	UK	UK	18	

*Kd* Carfilzomib-dexamethasone, *Vd* bortezomib-dexamethasone, *DVd* daratumumab- bortezomib-dexamethasone, *PVd* pomalidomide-bortezomib-dexamethasone, *Len-Cy-Pred* lenalidomide-cyclophosphamide-dexamethasone, *D-Kd* daratumumab-carfilzomib-dexamethasone, *D-Pd* daratumumab-pomalidomide-dexamethasone, *Isa-Pd* isatuximab-pomalidomide-dexamethasone, *KPd* carfilzomib-pomalidomide-dexamethasone, *Len-refractory* lenalidomide-refractory, *PFS* progression-free survival, *UK* unknown

### Outcomes of patients progressing on frontline lenalidomide therapy: data from phase 3 trials

(Table 1) For a patient progressing on R(d) as frontline therapy, the logical approach is a switch in the class of agent, from an IMiD to a PI. Bortezomib-dexamethasone (Vd) was the first combination used in this setting, resulting in a PFS ranging from 8 to 10 months^18^. Cyclophosphamide may also be added (VCd) to increase the response rate, but no prospective comparison of Vd versus VCd in relapse is available. The toxicity of Vd is well-known, and despite the subcutaneous or weekly administration of bortezomib, peripheral neuropathy remains the most important side-effect of this combination. Several phase 3 trials have evaluated PI-based combinations using Vd as control arm in RRMM and although it would have been desirable to know the efficacy of these combinations in lenalidomide-refractory patients, the reality is that only few truly lenalidomide-refractory patients were included. Four trials may be discussed in this setting: First, the phase 3 randomized ENDEAVOR study prospectively compared Vd versus carfilzomib-dexamethasone (Kd) until progression in the relapse setting in patients with 1 to 3 prior lines of therapy^19,20^. This study, a head-to-head comparison of two PIs, demonstrated that both PFS (median 18.7 months vs 9.4)^19^ and OS (median 47.6 vs 40)^20^ were superior with Kd across the whole group of patients. In this trial, the number of patients refractory to lenalidomide regardless of the number of prior lines of therapy was actually small, 51 in the Kd arm and 45 in the Vd arm, and the exact number of patients progressing on frontline lenalidomide is unknown^21^. The median PFS for the group of lenalidomide-refractory patients was rather short, 8.6 and 6.6 months with Kd and Vd, respectively^21^. These findings suggest that lenalidomide-refractory patients may not benefit as much from Kd as patients who respond well to prior lenalidomide (although in both cases, outcomes were better for Kd than for Vd). No OS data are available for lenalidomide-refractory patients treated with either combination. Of note, the schedule of Kd is more demanding than that of Vd, with intravenous administration of carfilzomib at the dose of 56 mg/m^2^ on days 1, 2, 8, 9, 15, and 16 in 28-day cycles until progression. The safety profile is also different from that of Vd, with fewer cases of PN, but higher rates of hypertension, dyspnea, cardiac failure and acute renal failure instead. Nevertheless, the rates of treatment discontinuation due to adverse events were identical in the two arms of the study.

Recently, Vd was compared to Vd plus daratumumab (DVd) in patients with relapsed MM who had received at least one prior line of therapy (CASTOR trial)^22^. The triplet combination was associated with a significant PFS improvement (median not reached versus 7.2 months, Hazard Ratio, HR, 0.39)^22^, which was confirmed in an updated analysis, in which, after a median follow-up of 19.4 months, the median PFS for DVd was 16.7 versus 7.1 months for Vd alone, HR 0.31^23^. As in the ENDEAVOR study, the number of patients progressing on frontline lenalidomide is unknown. The only information available is based on a sub-analysis, showing that patients refractory to lenalidomide, regardless of the number of prior lines of therapy, (DVd, n = 60; Vd, n = 81) also achieved a significant PFS benefit with DVd versus Vd, with medians of 7.8 versus 4.9 months, respectively, not very different from the data reported in the ENDEAVOR study for a similar subgroup of patients^24^. Overall survival data for this subgroup of patients are not available to date. Importantly, the safety profile of the triplet combination is acceptable, and daratumumab was not found to add any significant toxicity to the Vd combination.

The phase 3 Panorama 1 study, comparing Vd vs Vd+ panobinostat, enrolled a subgroup of patients progressing on lenalidomide as frontline therapy, but the number of patients in this setting was very small and prior treatment with lenalidomide was not a stratification factor^25^. Overall, the study showed that the combination of Vd+ panobinostat improved PFS by four months, but did not result in an OS benefit^26^. The toxicity observed in the panobinostat arm of the trial, especially the high frequency of grade 3–4 gastro-intestinal adverse events, fatigue and thrombocytopenia, does not argue in favor of the use of this triplet combination in lenalidomide-refractory patients.

Most recently, the phase 3 OPTIMISMM trial was conducted in patients with relapsed and/or refractory MM (median two prior lines of treatment). In this trial, the combination of pomalidomide, bortezomib and dexamethasone (PVd) demonstrated a significantly improved PFS (11.20 vs 7.10 months, HR 0.61) and a manageable safety profile compared to Vd^27^. This trial is different to those reviewed previously because it is the first and only phase 3 randomized trial, in which all patients had previously received lenalidomide. Moreover, 70% of patients were lenalidomide-refractory and among these, a significant number had received lenalidomide as first-line therapy and received either PVd (57.7%) or Vd (56.5%) as first rescue treatment. In an exploratory analysis, which compared the efficacy and safety of PVd vs Vd in patients with lenalidomide-refractory and lenalidomide-non refractory disease treated after one prior line of therapy, PVd was shown to reduce the risk of progression or death regardless of lenalidomide-refractoriness^28^. The median PFS was 17.84 months in the PVd arm (n = 64) vs 9.49 in the Vd arm (n = 65) for patients with lenalidomide-refractory disease after one prior line of treatment. This regimen, effective and tolerable in patients, for whom lenalidomide is no longer a treatment option, is not yet approved, but represents an interesting option at first relapse following lenalidomide, although OS data are lacking due to the relatively short follow-up (16.4 months)^28^.

Overall, the analyses of these four phase 3 trials show that few patients refractory to frontline lenalidomide therapy have been evaluated to date. This is not unexpected for PANORAMA-1, CASTOR and ENDEAVOR, which were designed before frontline lenalidomide given until progression became a frequently used treatment strategy, especially in Europe. The OPTIMISMM study, the last to be reported, which was intended to include patients previously exposed to lenalidomide, is closer to the real life setting and PVd therefore represents a realistic and effective combination. DVd and Kd, approved in RRMM, are also feasible, although their efficacy in patients progressing on frontline lenalidomide remains to be clarified. Vd-panobinostat would represent the last choice considering both efficacy and safety.

### Outcomes of patients progressing on frontline lenalidomide therapy: data from phase 1b/2 trials

As previously stated, there is a lack of phase 3 trial data in RRMM that can be used to make treatment decisions in patients progressing on frontline lenalidomide therapy, mainly because those trials were conducted before lenalidomide became widely used as continuous therapy in frontline (Table 1). However, there is some data regarding this particular group of patients from phase 2 trials, which evaluated new combinations based on PIs and/or pomalidomide +/− MoAbs. Major limitations are the small number of patients, as well as the short follow-up, and lack of OS data.

Jakubowiak et al. reported data of a phase 2 randomized trial comparing Vd versus Vd+ elotuzumab in 152 patients with RRMM showing a PFS benefit of the triplet combination in the intent-to-treat population (9.7 vs. 6.9 months)^29^. Sixty-six percent of the patients were treated at the time of the first relapse, but the number of cases progressing on lenalidomide is not reported, and a subgroup analysis of patients previously treated with IMiDs showed no PFS benefit of the addition of elotuzumab to Vd (HR: 0.87, 95% CI: 0.56–1.34)^29^.

The CHAMPION-1 trial evaluated the more convenient weekly administration of carfilzomib in combination with dexamethasone in RRMM^30^. In the phase 2 portion of the study, 31 / 89 patients (35%) treated at the dose of 70 mg/m^2^ carfilzomib were refractory to lenalidomide (not all of them at first relapse), and the response rate was 71%, very similar to the 74% ORR observed for the whole group of patients. The median PFS was 12.6 months for 104 patients treated at the maximum tolerated dose of 70 mg/m^2^ (phase 1 and 2), but the median PFS for patients refractory to frontline lenalidomide was not reported. A recent analysis was conducted on all patients, who had progressed on frontline lenalidomide therapy and were treated with Kd in both the ENDEAVOR (bi-weekly carfilzomib) and CHAMPION-1 (weekly carfilzomib) trials. The number was small (only 32 patients were identified overall), and the promising median PFS of 15.6 months requires confirmation in future studies^31^.

Carfilzomib and dexamethasone have been combined with daratumumab (D-Kd) in the phase 1b MMY1001 study^24^. Carfilzomib was used weekly at 70 mg/m^2^ plus low-dose dexamethasone, and the dose of daratumumab was 16 mg/kg QW on cycles 1–2, Q2W on cycles 3–6, and Q4W thereafter until progression. The trial included 85 patients, with 51 of them (59%) having lenalidomide-refractory disease (median two prior lines of therapy). The ORR was 79% for lenalidomide-refractory patients, and the 12-month PFS rate was 65% in this cohort (median follow-up 16.6 months)^24^, while OS data are not yet available. In the group of lenalidomide-refractory patients, only six were refractory to frontline lenalidomide. Therefore, the promising PFS achieved with this D-Kd regimen needs to be confirmed in our population of interest.

In the same MMY1001 trial, another arm tested the combination of pomalidomide-dexamethasone plus daratumumab (D-Pd)^24^. Ninety-two patients out of 102 enrolled into this arm were lenalidomide-refractory. The ORR for the whole group of patients was 66%, and the median PFS was 10.1 months after a median follow-up of 28.1 months. However, the number of patients progressing on frontline lenalidomide therapy included in this arm is unknown. The same combination, D-Pd, has been tested in 112 patients progressing after lenalidomide-based therapy (median two prior lines of therapy), of whom 84 (75.0%) were refractory to lenalidomide, in another phase 2 MM-014 trial conducted in North America (median follow-up 8.2 months)^32^. The ORR (primary end-point) was 75% in lenalidomide-refractory patients, and the 9-month PFS rate was 86.3% (range 76.5%-92.2%), while the median PFS was not estimable^32^. Here again, data focusing on patients with disease that is refractory to frontline lenalidomide are not available. Pomalidomide-dexamethasone has also been combined with MOR202, another MoAb targeting CD38, in a phase 1/2a study involving 21 patients (median three prior lines of therapy), with a median PFS of 17.5 months. However, there were only very few patients, who were treated with this combination for first relapse after frontline lenalidomide, and results in this subgroup of cases are not available^33^.

There are other pomalidomide-dexamethasone-based combinations that have been evaluated in populations that included patients with lenalidomide-refractory disease, but patients, who had received just one prior line of therapy, were not allowed to be included. These studies can therefore not be used to answer the question about the optimal combinations to rescue patients progressing on frontline lenalidomide therapy. Nevertheless, we briefly describe their results here because they will potentially be tested in the future in the population that is the subject of this manuscript. Pomalidomide-dexamethasose plus isatuximab, a third MoAb targeting CD38, has been evaluated in a phase 1b study including 45 patients with RRMM, 37 (82%) of whom were lenalidomide-refractory and had advanced disease with at least 2 prior lines of therapy (median 3). The combination resulted in a promising response rate and PFS (median 17.6 months for the whole group of patients)^34^. Another potential combination is pomalidomide-dexamethasone plus elotuzumab, which was tested in a phase 2 randomized trial versus pomalidomide-dexamethasone in patients mostly refractory to lenalidomide and PIs. However, this ELOQUENT-3 study, which showed improved PFS results in the elotuzumab arm (median 10.3 months) enrolled only patients who had received at least 2 prior lines of therapy, and no patients progressing on frontline lenalidomide were included^35^.

Finally, over the last few years, some trials that are being planned in the population of patients with newly diagnosed MM also include designs to evaluate rescue strategies for these patients. In line with this, pomalidomide has been combined with twice-weekly 56 mg/m^2^ carfilzomib and dexamethasone (KPd) in the prospective EMN011/HO114 trial conducted by the European Myeloma Network^36^. This phase 2 trial was designed for patients with refractory disease or first progression after having received therapy as part of the EMN02 trial, in which patients were randomized to frontline ASCT versus no frontline ASCT followed by consolidation and lenalidomide maintenance until progression. The analysis of the first 60 patients, 57 (95%) of whom had progressed on lenalidomide maintenance, was presented at the 2018 meeting of the American Society of Hematology. After four 28-day cycles of re-induction with KPd, patients were offered either salvage ASCT, if they had not received frontline intensive therapy, or four additional cycles of KPd (8 KPd cycles overall). Subsequently, patients with stable disease or better received pomalidomide 4 mg with or without dexamethasone in 28 days cycles until progression^36^. Responses to KPd were quick, with a median time to best response of two months. The toxicity of KPd was manageable, and at a median follow-up of 16.3 months, the median PFS was 18 months, with a better outcome in patients with standard-risk cytogenetics (HR = 0.27) and in patients, who had not received frontline ASCT (n = 25, HR = 0.49).

There are other new agents, which have been tested in combination with Vd in phase 1b/2 trials in RRMM, such as Vd+ venetoclax^37^, or Vd+ selinexor^38^, but no data are available in the specific subgroup of patients progressing on frontline lenalidomide.

Overall, the analyses of these different phase 2 trials show that only few patients refractory to frontline lenalidomide therapy can be evaluated, however, promising results were achieved with the combination of carfilzomib or pomalidomide plus a MoAb targeting CD38, or with the combination of carfilzomib plus pomalidomide.

### Important ongoing phase 3 trials in lenalidomide-refractory patients

Some of the phase 2 trials discussed above are the basis for important ongoing phase 3 studies in RRMM that will help in the identification of the best options for patients progressing on frontline lenalidomide (Table 2). These trials are comparing Kd +/− antiCD38 monoclonal antibodies, Pomalidomide-dexamethasone +/− antiCD38 monoclonal antibodies, Vd +/− venetoclax or Vd +/− selinexor and the first results are expected in 2019. Nevertheless, the limitation is that not only patients with disease refractory to frontline lenalidomide are included and the trials will therefore provide only subgroup analyses.

**Table 2:** Primary end-point, main inclusion criteria and estimated enrollment of ongoing phase 3 trials that include patients progressing on lenalidomide

CANDOR (NCT03158688):
Objective:
-To compare Kd, and Daratumumab-Kd in terms of PFS in patients with MM who have relapsed after 1 to 3 prior therapies.
Main inclusion criteria:
-Relapsed or progressive MM after last treatment
-Received at least 1, but not more than 3 prior lines of therapy for MM
Estimated enrollment: 466 patients
IKEMA (NCT03275285):
Objective:
-To compare Kd, and Isatuximab-Kd in terms of PFS in patients with MM who have relapsed after 1 to 3 prior therapies.
Main inclusion criteria:
-Patients with MM previously treated with prior 1 to 3 lines
Estimated enrollment: 300 patients
ICARIA (NCT 02990338):
Objective:
-To compare Pom-dex, and Isatuximab-Pom-dex in terms of PFS in patients with RRMM
Main inclusion criteria:
-Patients must have received at least 2 prior lines of anti-myeloma therapy
-Patients must have failed treatment with lenalidomide and a proteasome inhibitor
-Patients must have progressed on or within 60 days after end of previous therapy before study entry, i.e., refractory to the last line of treatment
Estimated enrollment: 300 patients
APPOLLO / EMN14 (NCT 03180736):
Objective:
-To compare Pom-dex, and Daratumumab-Pom-dex in terms of PFS in patients with RRMM
Main inclusion criteria:
-Subjects must have received prior anti-myeloma treatment. The prior treatment must have included both a PI- and lenalidomide-containing regimen. The subject must have had a response to prior therapy
-Subjects must have documented evidence of PD on or after the last regimen
-Subjects who received only 1 line of prior treatment must have demonstrated PD on or within 60 days of completion of the lenalidomide-containing regimen
Estimated enrollment: 302 patients
BOSTON (NCT03110562):
Objective:
-To compare Vd, and Vd-Selinexor in terms of PFS in patients with RRMM
Main inclusion criteria
-Patients must have received at least 1 prior anti-MM regimen and no more than 3 prior anti-MM regimens
-Documented evidence of progressive MM on or after their most recent regimen
Estimated enrollment: 364 patients
BELLINI (NCT02755597):
Objective:
-To compare Vd-placebo, and Vd-Venetoclax in terms of PFS in patients with RRMM
Main inclusion criteria
-Participant has documented relapsed or progressive MM on or after any regimen, or refractory to the most recent line of therapy
-Participant must have received prior treatment with at least one, but no more than three prior lines of therapy for MM
Estimated enrollment: 291 patients

*Kd* carfilzomib-dexamethasone, *Pom-dex* pomalidomide-dexamethasone, *Vd* bortezomib-dexamethasone

## Conclusion

The number of patients treated with frontline lenalidomide that will progress while on therapy is likely to increase markedly in the coming years. To date, very few trials have targeted this patient population in order to define the optimal salvage regimen. The available results on approved combinations are restricted to a very low number of patients and show sub-optimal outcomes. Moreover, no OS data have been published to date.

The most encouraging data are those achieved with the combinations of PVd or KPd, which are not approved. The results of ongoing trials using Kd or Pomalidomide-dexamethasone plus antibodies targeting CD38 are eagerly awaited.

We should also encourage companies and leading authors of important trials, in which frontline therapy with lenalidomide was used either as low-dose single agent (lenalidomide maintenance after ASCT^9–12^) or at the dose of 25 mg per day with dexamethasone (for example, continuous lenalidomide-dexamethasone in the FIRST trial^7^, SWOG S0777^8^, or ECOG-E4A03^39^) to report on the results of the salvage therapies that patients received at the time of progression, even if those salvage regimens were not preplanned in the study. This represents a large amount of data, that are probably not collected yet, but that may help to solve the challenging issue of the optimal rescue therapies after frontline lenalidomide failure.
